# Click to consult: psychiatrists’ perspectives on how telepsychiatry impacts communication in the emergency department

**DOI:** 10.3389/fpsyt.2025.1629475

**Published:** 2025-09-05

**Authors:** Ligat Shalev, Maram Khazen, Adam J. Rose, Gadi Lubin, Renana Eitan

**Affiliations:** ^1^ School of Public Health, Hebrew University of Jerusalem, Jerusalem, Israel; ^2^ The Max Stern Department of Health Systems Management, Yezreel Valley College, Yezreel Valley, Israel; ^3^ The Jerusalem Mental Health Center, Jerusalem, Israel

**Keywords:** organizational innovation, telepsychiatry, telehealth, emergency department, implementation science, digital health services

## Abstract

**Background:**

Telepsychiatry, the use of video-calls for evaluation, treatment, or follow-up, is increasingly integrated into psychiatric care. However, research on its impact remains limited, particularly in emergency department (ED) settings where it is used for evaluations considering involuntary psychiatric hospitalization. Little is known about how telepsychiatry influences psychiatrist–patient interactions and the dynamics between attending and resident psychiatrists. This study explores how the transition to telepsychiatry, with the attending psychiatrist evaluating patients remotely while the resident and patient remain in the ED, affects communication and interaction as reported by physicians.

**Methods:**

This qualitative study involved semi-structured interviews with all 36 psychiatrists, including attendings and residents, working in the ED of a psychiatric hospital. Thematic analysis and explanatory content analysis were used. The initial analysis was conducted by one researcher, with two additional researchers independently reviewing the data to ensure trustworthiness.

**Results:**

Three major themes emerged. The first theme focused on changes in psychiatrist–patient communication. Using the CanMEDS Communicator Role framework, we examined how communication patterns shifted with the transition from in-person evaluations to telepsychiatry. Concerns were raised about whether telepsychiatry weakens professionalism and the therapeutic connection between psychiatrists and patients. However, most interviewees considered telepsychiatry sufficient for assessing the need for involuntary hospitalization. The second theme examined residents’ evolving role in telepsychiatry, which has increased their responsibility as mediators in patient evaluations. Without attending psychiatrists physically present, residents must relay crucial sensory and contextual details (e.g., non-verbal cues, odors, prior events) enhancing their clinical judgment and communication skills. The final theme addressed shifts in the attending-resident dynamic. The remote nature of telepsychiatry places emphasis on the degree of trust between attending psychiatrists and residents. Some attendings expressed confidence in their residents’ evaluations, while others raised concerns about potential biases that may arise when attending psychiatrists rely on second-hand information instead of conducting evaluations themselves.

**Conclusions:**

The shift to telepsychiatry changed communication between psychiatrists, residents, and patients, bringing benefits and challenges. These findings underscore how changes in care delivery can impact communication dynamics. This emphasizes the need for telepsychiatry implementation to include ongoing evaluation and training, to maintain effective and ethical care.

## 1 Background

Effective communication and collaboration among healthcare teams is often thought of as an important predicate for delivering high-quality care (1, 2). Interprofessional collaboration, such as between physicians and nurses, has the potential to enhance the delivery of person-centered care and improves patient and system outcomes (3). Studies have shown that healthcare teams practicing collaboratively can reduce medical errors and increase patient safety (4, 5). Buljac-Samardzic and colleagues (6) conducted a systematic review of 297 studies on interventions to improve healthcare team effectiveness. The interventions included, for example, simulated training and educational seminars designed to enhance knowledge and improve the ability to work effectively within teams. They found that those interventions positively impact team performance and patient outcomes. Collaboration within interdisciplinary teams can occur under specific conditions.

Some theoretical models of information sharing and communication in healthcare settings emphasize the importance of building relationships founded on trust, mutual respect, and collaboration culture (7–9). For an interdisciplinary team, one of the core foundations of effective communication is mutual trust and respect, along with recognizing and valuing the contributions of other professionals (8). Engaging in a collaborative process requires professionals to embrace a certain level of interdependence and flexibility (10). Collaboration also requires professionals to integrate the expertise of various specialists into patient care plans and communicate their perspectives in an inclusive and participatory manner (10). However, medical teams often experience power dynamics and conflicts that influence team interactions (11). Moreover, teams that involve hierarchical interactions, meaning supervisor and supervisees, and where there is an inherent power gradient, may tend toward maladaptive communication patterns such as avoidance or obedience (12). This dynamic can appear in interdisciplinary teams, as well as in intraprofessional teams – teams from the same profession, such as attending and resident physicians.

Communication between attending and resident physicians is inherently hierarchical. The misuse of power within a hierarchical structure can negatively impact supervisees in general, and residents in particular, with especially strong effects on the learning process and professional communication (13–16). Looman and colleagues (7) conducted 45-hour observations and 42 interviews with residents in five Dutch hospitals, examining resident interactions in emergency departments (ED). They found that while hospitals provide opportunities for collaborative learning, these are rarely utilized due to barriers such as power dynamics, and specifically lack of respect and inherent inequality. As previously mentioned, respect is a fundamental aspect of effective communication and collaboration between team members, and its absence can significantly hinder communication (7–9).

There are characteristics that have been suggested to define the “good” resident in hierarchal relationships with specialist. An Australian research group conducted 20 interviews with attending and resident surgeons across eight surgical specialties to explore what defines a “good” resident. One key theme that emerged from both attending and resident surgeons was demonstrating strong teamwork skills. The ability to put one’s trust in the resident was another theme mentioned by attending surgeons (17). This component is considered as a basic requirement for good communication among team members (8). There are relatively few studies on the characteristics of a “good” psychiatric resident. A 2009 study in Singapore used a focus group with senior psychiatrists to explore the qualities of a good psychiatrist and resident. Based on the focus group results, the researchers created a survey and administered it to 74 psychiatry trainees and attending psychiatrists. One key finding was that positive relationships with colleagues and patients are essential to a high-quality training program (18). Similarly, a 1997 study examined whether psychiatry residents and program directors agree on the key factors determining residency program quality. The study found that residents placed greater emphasis on interpersonal relationships, both with colleagues and patients, while program directors focused more on professionalism, knowledge, and institutional resources (19).

One strategy to improve communication among physicians, including those of different levels of seniority, is through brief training sessions. For example, Frank and colleagues (20) developed a 90-minute interactive workshop on care transitions in psychiatry for residents and fellows. Participants analyzed patient cases across four care settings, addressing logistical and communication challenges. Survey results showed high participant satisfaction, with 90% identifying key handoff elements and 83% understanding patient transfer logistics. Besides communication with team members, communication with patients is also a skill that residents are required to acquire (21).

Effective communication with patients is a critical skill that residents must develop to ensure high-quality care (21). Castillo and colleagues (22) examined communication differences between US psychiatric residents and attendings during medication-management appointments. Residents had longer visits, prioritized relationship-building, and sounded friendlier, while attending psychiatrists focused on biomedical data and appeared more dominant in the interaction. Yet, studies show that even residents, from psychiatry and other fields, need to improve skills for communicating with patients (23). A cross-sectional survey conducted in India explored the barriers residents face in developing effective communication skills. The study identified lack of time, long working hours, and insufficient communication training as the primary obstacles to developing effective communication (23). Structured communication skill training for residents has been shown to increase patient satisfaction and improve health outcomes (21). The need for training in communication, empathy, and active listening is essential for residents across all medical fields, but especially for psychiatry residents due to the mental health challenges of their patients (24, 25). Communication, empathy, and active listening are also crucial for resident psychiatrists, as psychiatric diagnoses rely primarily on patient-reported information rather than physical exams or tests (24). But while excellent communication is arguably more critical in psychiatry than in any other field of medicine, communication skills are under-emphasized in the curriculum of psychiatric residency training programs, and variability across training programs poses a challenge for assessing their effectiveness (26).

There is evidence that even brief training sessions can produce demonstrable changes in communication. For example, Amsalem and colleagues (27) evaluated the effectiveness of a five-hour workshop using a standardized patient-based training module to enhance Israeli psychiatric residents’ communication skills in delivering difficult news. In this study, residents’ measured communication skills and self-reported confidence both improved. In other studies, communication skills training produced other desirable changes, such as increased empathy, but not improved communication itself, hinting that the “dose” of training required to actually improve communication may be quite high. Noordman and colleagues (28) successfully improved empathy scores among Dutch medical residents, as reported by patients, through a three-day training program. However, the training failed to significantly enhance residents’ communication skills. Similarly, an Iranian research group examined the effectiveness of communication skills training as a distance learning method for improving empathy among first-year psychiatry residents. Residents who attended the full two-day workshop showed a significant increase in empathy, while those in the distance learning group (who only watched a recording of the first day) showed no improvement (29).

Effective communication between residents and attending physicians is crucial after hours, meaning evening and night shifts. However, we have only limited data regarding such interactions, especially in the setting of psychiatric emergency care. One study found that attending physicians expected pediatric residents to communicate more frequently and promptly after hours than the residents themselves anticipated, especially in cases that were ambiguous or evolving (30). Similarly, another study comparing surgical and medical specialties found that while communication occurred in 84% of night shifts, attending physicians initiated contact about half as often as professional guidelines recommended (31).

We only found one paper that addressed interaction expectations between attending and resident physicians in an emergency psychiatry setting. In this study, attending psychiatrists preferred greater involvement in overnight cases than the residents would have preferred, especially for cases involving staff injuries, patient drug use, and conflicts with attending psychiatrists from the department of emergency medicine (32). While psychiatry attendings favored increased involvement during overnight hours, a randomized controlled trial conducted in non-psychiatric departments showed that greater supervision, implemented by having attending psychiatrists join work rounds on previously admitted patients, did not reduce medical errors but did decrease resident autonomy and efficiency. In the increased supervision group, interns spoke significantly less during rounds than in the control group (55 minutes vs. 64 minutes; p=0.008), and residents reported feeling less efficient (55% vs. 73%; p=0.02) and less autonomous (58% vs. 97%; p<0.001) when attending psychiatrists were present (33).

In addition to interactions among the medical staff, interactions between health workers and patients are also important. Navas and colleagues (34) reviewed interpersonal factors affecting both patient and provider experiences in psychiatric EDs. Patients valued clear communication, but often reported receiving poor explanations, lack of empathy, and being stigmatized, especially during encounters involving restraints or involuntary hospitalization. On their side, providers struggled to communicate effectively, particularly with patients experiencing suicidality or substance use disorders. These findings highlight misalignment between patient expectations and provider communication styles, and emphasize the need for improved communication strategies, cultural sensitivity, and patient-centered approaches in psychiatric EDs.

In recent years, psychiatry services have increasingly adopted telepsychiatry, which involves evaluation and treatment via live video (35, 36). While telepsychiatry is used in emergency settings, our group conducted a scoping review in 2024 on its use in the ED. We found only 12 papers reporting data on this practice in the ED, and of those, only two focused on its utilization for evaluating patients for potential involuntary admission (37). One study, conducted by our group, assessed the validity and accuracy of telepsychiatry evaluations for possible involuntary evaluations in comparison to in-person evaluations (38). Another study examined the cost-effectiveness of using telepsychiatry to facilitate the conversion of involuntary commitments into voluntary hospitalizations (39).

As a new modality, telemedicine in both general medicine and psychiatry has limited studies integrating it into the curriculum for residents working in the ED and other healthcare settings (40, 41). Incorporating telehealth education into medical curricula has been shown to improve participants’ knowledge and skills (42). However, existing curricula lack consistency, with significant variation in content (42, 43). Curricula specifically addressing telehealth in the ED are even more limited, with only one study found focusing on training for telehealth use in the ED (44). Additionally, no studies were found to explore communication between the psychiatrists and the patients for cases of possible voluntary admissions.

Regarding the need for data on the use of telepsychiatry in the ED for considering involuntary admissions, more research is needed on communication that involves hierarchical relationship such as those between attending and resident psychiatrists, particularly in psychiatric EDs. This study aims to explore the impact of telepsychiatry on interactions and communication between attending psychiatrists, resident psychiatrists, and patients in the ED. It is part of a broader study evaluating the effectiveness and implementation of live-video consultations for involuntary admission assessments during evening and night shifts, with the resident and patient located in the hospital while the attending physician is remote (45). Preliminary findings, based on objective measures such as the rate of involuntary admissions and potential clinical decision errors, suggest that live-video consultations produce clinical outcomes comparable to face-to-face evaluations.

This study aimed to gain a deeper understanding of how the transition to telepsychiatry, rather than the attending physician being physically present, influenced interaction and communication between psychiatrists and patients from the physicians’ perspective, as well as the interaction and communication between attending and resident psychiatrists in the psychiatric ED.

## 2 Materials and methods

### 2.1 Study design, setting and participants

This qualitative study is part of a larger project on implementing telepsychiatry in EDs (45). In Israel, patients who are considered for involuntary hospitalization must be evaluated in person by an attending psychiatrist. For those patients, if the attending psychiatrist finds that the patient poses a risk to themselves or others and does not consent to hospitalization, the case is presented by phone to the district psychiatrist, who can approve or decline the involuntary hospitalization. In our study, the procedure was conducted similarly, except that during hours when attending psychiatrists are not ordinarily present (i.e., weekends, holidays, and from 3 PM–7 AM), the attending psychiatrist’s evaluation of the patient was performed via a video-link. This means that after the patient was examined by the resident and presented to the attending psychiatrist, the patient is taken to a private room with a computer, where a secure video connection is established between the resident and the patient on one end and the attending psychiatrist on the other. Based on the resident’s presentation of the patient and the attending psychiatrist telepsychiatry evaluation, the attending psychiatrist determines whether involuntary admission is necessary, relying on the information provided by the resident. The only change in the procedure is that instead of arriving in person, the attending psychiatrist conducts the exam via a secure video-chat, although he or she is able to come to the ED physically if the remote evaluation does not seem adequate. The psychiatric team participated in a 1.5-hour training session covering the study’s goals, the adjustments in evaluations, and the documentation when conducting evaluations via telepsychiatry. Additionally, they were encouraged to reach out with any questions or concerns regarding the new approach to care delivery. Because telepsychiatry has not previously been used in Israel for this purpose, we received a temporary waiver from the Ministry of Health to allow remote evaluations during the study period for the purpose of evaluation (45).

The data for the present study comes from the ED located in a specialized psychiatric hospital in a large city with a population of 1 million. Unlike general hospitals, this psychiatric facility is dedicated solely to emergency psychiatric care and includes various specialized units, such as closed-door and open-door units, as well as units designed to address specific populations, such as individuals with comorbid schizophrenia and substance use disorders. The ED operates 24 hours a day, providing immediate care, directing patients for hospitalization either within the same hospital or elsewhere, or facilitating their release back to the community for continued care. We interviewed all attending and resident psychiatrists who have experienced telepsychiatry during evening and night shifts (3 PM to 7 AM) and work in person at the ED and hospital departments during regular working hours (7 AM to 3 PM). We chose to interview only psychiatrists, as they were the sole healthcare professionals directly involved in operating and utilizing telepsychiatry.

### 2.2 Data collection

In-depth semi-structured interviews were conducted in January 2025 with all attending and resident psychiatrists working in the ED. We selected this data collection method to capture retrospective psychiatric perspectives on telepsychiatry freely and without interruption. The lead researcher (LS) approached all the psychiatrists who work at the ED to participate in the study anonymously, and after agreeing they provided informed consent. They were asked about their views on the use of telepsychiatry in the ED, their interaction and communication with attending psychiatrists, and, for residents with over a year of experience, whether these interactions had changed after the implementation of telepsychiatry. Interviewees also provided background characteristics, such as age and seniority (see **Supplementary File 1**). The interviews were transcribed for analysis, and ethical approval was obtained from the Institutional Research Ethics Board (22-21; November 6, 2022).

### 2.3 Data analysis

Data was analyzed using two approaches. The first was explanatory content analysis (46) based on the CanMEDS Communicator Role framework, which outlines communication with patients by five key concepts (47, 48). The second analysis approach used was phenomenological qualitative analysis, using a multi-step process for extracting themes systemically from qualitative data. Exploratory analysis follows a multi-step process to systematically extract themes from qualitative data (49). In the first step, all qualitative material was read for the purpose of learning and in-depth understanding. After that, the data were coded, which refers to an orderly procedure of processing and sorting the material according to categories and themes, to reveal the recurring themes arising from the materials (50, 51). These two qualitative approaches were chosen as they are well-suited to the needs of a health services research study of this nature. The initial analysis was conducted by the lead researcher (LS), a PhD expert in social work and public health with specialization in mixed research methods. To ensure the reliability of the findings, two additional researchers – AJR, a mixed-methods researcher and physician, and MK, a PhD communication expert specializing in qualitative research and a practicing pharmacist – independently reviewed the analysis. Any discrepancies were resolved through further discussions until consensus was achieved. The researchers’ diverse backgrounds in qualitative research, mental health, and emergency psychiatric care enriched data analysis. To minimize bias, they employed independent coding, ensuring personal experiences did not unduly influence findings.

## 3 Results

Thirty-six semi-structured interviews were conducted with 19 attending psychiatrists and 17 resident psychiatrists. Among the attending psychiatrists, 58% were male, with an average age of 50.8 years; they had an average of 15.4 years of experience in psychiatry. Of the 17 resident psychiatrists, 76% were male, with an average age of 35.9 years; they had an average of 4.4 years in their residency. As this is the first time telepsychiatry is being used for potential involuntary admissions in Israel, none of the psychiatrists had prior experience with this specific application. However, some attending psychiatrists mentioned using video consultations for ongoing therapy with patients they had previously met in person (**Table 1**).

**Table 1:** Background characteristics of the attending psychiatrists (n=19) and the resident psychiatrists (n=17).

Participant characteristics	Attending psychiatrists (n=19)	Resident psychiatrists (n=17)
Sex, male (vs. female; No., %)	11, 58%	13, 76%
Age (years; Median, standard deviation)	50.8, 9.6	35.9, 10.3
Seniority working at the hospital (years; Max, min, M, SD)	15.4, 8.9 (Min=1, Max=33)	4.4, 4.9 (Min=0.5, Max=22)

Thematic analysis identified three major themes regarding the interaction between attending and resident psychiatrists using telepsychiatry in the ED. The first theme focuses on communication patterns between attending psychiatrists, resident psychiatrists, and patients, analyzed through the CanMEDS Communicator Role framework (47, 48). The second theme explores the evolving role of residents, particularly in terms of increased responsibility and expanded teaching opportunities with the implementation of telepsychiatry. The third theme examines changes in the attending–resident psychiatrist dynamic, highlighting shifts in their interaction due to telepsychiatry. Themes, sub-themes, and supporting quotes from the interviews will be presented in the following section.

### 3.1 Changes in patterns of physician-patient communication patterns

The introduction of telepsychiatry and video-based interactions changed the mode of communication between psychiatrists, regardless of their seniority, and their patients, as it was a new modality that altered the way psychiatrists interacted with patients. To structure our discussion of this topic, we will utilize the CanMEDS Communicator Role framework, which focuses on physician–patient interactions (47, 48). This framework highlights relationship-building, information exchange, and patient-centered care as key objectives of physician–patient communication. It ensures that physicians effectively engage with patients and their families through five essential concepts: 1. Building professional therapeutic relationships by fostering trust, demonstrating empathy, and tailoring communication to meet patient needs; 2. Collecting and synthesizing information using structured interviewing techniques that integrate patient perspectives; 3. Communicating healthcare information in a clear, accurate, and timely manner, including the transparent disclosure of medical errors; 4. Actively involving patients in care planning while respecting their cultural and personal preferences; 5. Maintaining and sharing medical records through accurate documentation, digital communication, and confidentiality, ensuring patient safety and continuity of care. Our analysis focuses on how telepsychiatry has influenced communication between psychiatrists, at all levels of seniority, and their patients (**Table 2**).

**Table 2:** Patient-doctor communication themes via telepsychiatry in the ED adapted to the CanMEDS framework, sub-themes and interview quotes.

Concept	Description	Sub-themes	Examples for interview quotes
1. Establishing professional therapeutic relationships	Building trust and empathy through personalized communication to foster professional relationships with patients	Concerns about professionalism while using telepsychiatry	“Telepsychiatry is an attempt to reduce the profession to its essence – gathering data to determine a diagnosis and treatment. However, in my view, the true essence lies in building a connection. The profession is drifting away from this, and telepsychiatry is a symptom of that shift” (Attending psychiatrist with over 20 years of experience)
		Quicker patient evaluations and care decisions	“Telepsychiatry only helped. We see patients more quickly, which allows for better collaboration with them – they are less agitated and frustrated from waiting. This improves patient experience. Someone waiting two hours just for an on-call psychiatrist’s evaluation- anyone in the ED could become aggressive or restless” (Attending psychiatrist with 5 years or less of experience)
2. Gathering and synthesizing information	Effective information gathering through structured interviews and incorporating patient and family perspectives	Most participants felt telepsychiatry was sufficient for gathering patient information and determining the need for involuntary hospitalization, though some expressed concerns it may be less effective than in-person evaluations	“A confused person who arrives with the police, for example, is extremely anxious. When they are asked if they’re willing to speak over video-call, the audio and video quality may be poor, making the situation even more confusing for everyone. It’s not effective – the human connection is missing in many ways, making it impossible to establish rapport. It feels like talking to a robot” (Attending psychiatrist with over 20 years of experience)
		Residents maintained primary responsibility for evaluating the patient’s condition under the supervision of the attending psychiatrist	“Telepsychiatry is completely reliable. You always know the limitations of the evaluation. Even in face-to-face assessments, there are limitations, such as partial patient cooperation. This is no different [in reliability] from an in-person evaluation” (Attending psychiatrist with 10–20 years of experience)
		Involuntary hospitalization decisions depend on the mental state exam, family and community support, and other factors affecting the patient’s potential well-being if discharged	“I was concerned that assessing a patient via video would be difficult, but in practice, it is possible to get a clear impression of the patient … My philosophy is that not every patient who arrives at the ED in a psychotic state necessarily requires an involuntary hospitalization order. Factors such as the family’s ability to manage the situation, the treating physician’s clinical approach, previous hospitalization history, and the patient’s level of cooperation all play a role. The decision is not purely clinical but also considers broader contextual factors. I can’t say that the evaluation is identical to an in-person assessment, but it’s good enough” (Attending psychiatrist with 10–20 years of experience)
3. Sharing healthcare information + 4. Engaging patients in care planning	Sharing clear, timely healthcare information with patients and families, including diagnoses, treatment options, risks, and errors. Also, engaging patients in care planning by respecting their preferences and guiding self-care through health technologies	Most participants found telepsychiatry evaluations comparable to in-person ones. However, one resident expressed frustration when the attending psychiatrist logged off, leaving them to deliver decisions to dissatisfied patients	“The attending psychiatrist is the one making the decisions and recommendations, and it feels wrong that they are not the one communicating those decisions directly to the patient. The attending psychiatrist should spend an additional 5–10 minutes talking to the patient – that part is missing. I feel like a broken telephone at times. If the attendings psychiatrists were the one delivering the decision, it would resolve the situation more effectively” (Resident psychiatrist with more than two years of experience)
5. Documenting and sharing medical information	Accurate record-keeping, secure digital communication, and confidentiality to ensure patient safety, care continuity, and regulatory compliance	Most participants noted that documentation remained similar to in-person evaluations, with residents drafting discharge papers under the attending’s supervision. However, telepsychiatry introduced challenges, such as residents needing to log out when attendings accessed patient files remotely, and spending time troubleshooting or repeating missed sentences, which disrupted the workflow	“I have to exit the patient’s file so the attending psychiatrist can log in, and I can’t type during the evaluation because I’m sitting next to the patient and worried, they might do something to the computer – they could easily break it. I also serve as an intermediary. The patient says, “I didn’t hear what you said,” so I have to relay information between the attending [psychiatrist] and the patient. Or the attending psychiatrist didn’t hear, so I have to repeat it to them. Because of this, I can’t type in the patient’s file during the session, since I constantly need to mediate the conversation, which demands my full attention. It’s very convenient for the attending psychiatrist but very frustrating for the resident…. For me as the resident, it just creates more chaos” (Resident psychiatrist with more than two years of experience)

#### 3.1.1 Establishing professional therapeutic relationships

This communication concept emphasized the importance of building professional relationships with patients by fostering trust, empathy, and tailoring communication to their individual needs.

A key theme that arose from the interviews was professionalism. Medical professionalism reflects a commitment by healthcare providers to uphold shared competency standards and ethical principles, ensuring high-quality care for patients and the public (52). Many participants voiced concerns about whether telepsychiatry compromises professionalism by leading to superficial or impersonal assessments, particularly in cases involving involuntary hospitalization. While both attending psychiatrists and residents acknowledged telepsychiatry’s efficiency, opinions varied on its alignment with professional standards. Some participants believed that, despite its limitations, telepsychiatry provided a sufficient and comparable alternative to in-person evaluations for determining the need for involuntary hospitalization. Others, however, argued that in-person evaluations foster a stronger therapeutic connection with patients. Additionally, some participants noted that the resident’s physical presence during an assessment helps establish a meaningful patient connection, allowing the attending psychiatrist’s remote evaluation to maintain its professional integrity.

“If a patient is agitated and violent, I use my judgment even for in-person evaluations, relying on previous hospitalizations, police reports, and family accounts. But with telepsychiatry, I can do this in a very similar way. If a patient turns off the computer, there’s nothing I can do, whereas in the ED, I can try to restart the conversation. The final outcome wouldn’t change if the patient is psychotic, cursing at me, and has attacked their family. However, my ability to establish a connection, persist, and conduct a deeper evaluation is somewhat compromised in telepsychiatry. That said, I don’t think this is crucial or significantly impacts decision-making in the ED” (Attending psychiatrist with 10–20 years of experience)

“The downside of telepsychiatry is the lack of direct human interaction, but when a resident is present, it doesn’t have the same impact. There is someone physically with the patient to explain and guide them through the process” (Resident psychiatrist with two years or less of experience)

Telepsychiatry facilitated quicker patient evaluations and care decisions, leading most attending psychiatrists and residents to regard it as an enhancement to the therapeutic process. By reducing wait times, enabling patients to receive care before their mental state worsens, and improving their overall experience in the ED, telepsychiatry was widely perceived as a valuable asset in emergency psychiatric care.

“Telepsychiatry only helped. We see patients more quickly, which allows for better collaboration with them – they are less agitated and frustrated from waiting. This improves patient experience. Someone waiting two hours just for an on-call psychiatrist’s evaluation – anyone in the ED could become aggressive or restless” (Attending psychiatrist with 5 years or less of experience)

Some participants noted that telepsychiatry could be confusing for patients, especially when informed that the attending psychiatrist would evaluate them via video. According to the interviewees, some patients found it difficult to understand the need for an additional evaluation after already being evaluated by the resident. However, this dynamic may not significantly differ from the experience of being examined by two physicians in person rather in telepsychiatry.

#### 3.1.2 Gathering and synthesizing information

This concept emphasizes the collection and integration of information through effective interviewing techniques, clearly structured patient encounters, and the inclusion of relevant perspectives from patients and their families.

Some attending psychiatrists and residents expressed concerns that telepsychiatry evaluations may be less effective in gathering and synthesizing information compared to in-person evaluations, resulting in lower confidence in telepsychiatry relative to traditional face-to-face evaluations.

“A confused person who arrives with the police, for example, is extremely anxious. When they are asked if they’re willing to speak over video-call, the audio and video quality may be poor, making the situation even more confusing for everyone. It’s not effective – the human connection is missing in many ways, making it impossible to establish rapport. It feels like talking to a robot” (Attending psychiatrist with over 20 years of experience)

However, most interviewees felt that telepsychiatry did not significantly impact the process of gathering and synthesizing information about a patient’s psychological state, or at the very least, was sufficient for determining the need for involuntary hospitalization. In both telepsychiatry and in-person evaluations, the process typically follows the same structure: the resident conducts the initial interview with the patient, their family, and/or relevant community members, such as therapists. The resident then presents their findings to the attending psychiatrist, who asks probing questions – both to guide the learning process and to clarify key points. Only after this step does the attending psychiatrist interview the patient directly. Many participants perceived this workflow as largely unchanged in telepsychiatry, with residents continuing to take primary responsibility for assessing the patient’s condition under the supervision of the attending psychiatrist.

“Telepsychiatry is completely reliable. You always know the limitations of the evaluation. Even in face-to-face assessments, there are limitations, such as partial patient cooperation. This is no different [in reliability] from an in-person evaluation” (Attending psychiatrist with 10–20 years of experience)

“The telepsychiatry evaluation is completely reliable. While it’s true that you can’t smell or fully observe the extent of a patient’s neglect, for patients evaluated by an on-call [attending] psychiatrist [via video], psychotic content, hallucinatory thoughts, and thought process disturbances still come through clearly … The two main concerns were that evaluations would be less reliable and that patients might refuse or that their paranoia would be triggered. However, through my experience with telepsychiatry, I have found that both fears have faded” (Resident psychiatrist with more than two years of experience)

Moreover, some attending and resident psychiatrists emphasized that involuntary hospitalization decisions rely not only on the mental state examination but also on factors such as family and community support and other external conditions affecting the likelihood of a patient doing well if sent home. This viewpoint emphasizes that psychiatric evaluation is only one component of the broader decision-making process for hospitalization or discharge.

“I was concerned that assessing a patient via video would be difficult, but in practice, it is possible to get a clear impression of the patient … My philosophy is that not every patient who arrives at the ED in a psychotic state necessarily requires an involuntary hospitalization order. Factors such as the family’s ability to manage the situation, the treating physician’s clinical approach, previous hospitalization history, and the patient’s level of cooperation all play a role. The decision is not purely clinical but also considers broader contextual factors. I can’t say that the evaluation is identical to an in-person assessment, but it’s good enough” (Attending psychiatrist with 10–20 years of experience)

#### 3.1.3 Sharing healthcare information +3.1.4 engaging patients in care planning

The third CanMEDS concept emphasizes the importance of sharing healthcare information with patients and their families in a clear, accurate, and timely manner, ensuring they understand diagnoses, treatment options, and potential risks – including the transparent disclosure of medical errors. Closely related is a fourth concept, which highlights the importance of engaging patients in care planning by fostering open discussions, respecting cultural and individual preferences, and guiding them in using health technologies to enhance self-care.

In the ED, evaluations for involuntary hospitalization typically lead to a decision to either admit or discharge a patient. Given the fast-paced nature of the ED, interactions with patients are naturally more limited compared to community or inpatient settings. However, efforts are made to provide explanations to patients and families to minimize the need for forced hospitalization. A shortened evaluation by the attending psychiatrist, particularly in telepsychiatry, may reduce communication and limit the extent of explanations and engagement regarding hospitalization or discharge decisions.

As noted earlier, most attending psychiatrists and residents considered telepsychiatry evaluations to be comparable to in-person evaluations, suggesting that information transfer to patients and families remains sufficient. Interestingly, while many participants focused on whether video-based evaluations were “good enough” for gathering information from patients, fewer comments were made about the effectiveness of conveying information from the physician to the patient. Only one remark, from a resident psychiatrist with more than two years of experience, expressed frustration over cases where the attending psychiatrist logs off after the video evaluation, leaving the resident responsible for delivering hospitalization or discharge decisions to dissatisfied patients.

“The attending psychiatrist is the one making the decisions and recommendations, and it feels wrong that they are not the one communicating those decisions directly to the patient. The attending psychiatrist should spend an additional 5–10 minutes talking to the patient – that part is missing. I feel like a broken telephone at times. If the attendings psychiatrists were the one delivering the decision, it would resolve the situation more effectively” (Resident psychiatrist with more than two years of experience)

#### Documenting and sharing medical information

The final CanMEDS concept emphasizes the importance of proper record-keeping, digital communication, and confidentiality to ensure patient safety, continuity of care, and regulatory compliance.

This topic was raised by only one resident, with most participants noting that the documentation process remained largely unchanged from in-person evaluations. Typically, the resident drafts the discharge papers under the attending psychiatrist’s supervision, with the attending psychiatrist providing feedback, requesting revisions, and giving final approval.

However, one resident pointed out new challenges introduced by telepsychiatry. When the attending psychiatrist logs in remotely to access patient files, the resident must log out, preventing simultaneous documentation of the evaluation. Additionally, rather than focusing on documentation, residents often find themselves troubleshooting technical issues or repeating missed sentences for the attending psychiatrist or patient, further disrupting the workflow.

“I have to exit the patient’s file so the attending psychiatrist can log in, and I can’t type during the evaluation because I’m sitting next to the patient and worried, they might do something to the computer – they could easily break it. I also serve as an intermediary. The patient says, ‘I didn’t hear what you said,’ so I have to relay information between the attending [psychiatrist] and the patient. Or the attending psychiatrist didn’t hear, so I have to repeat it to them. Because of this, I can’t type in the patient’s file during the session, since I constantly need to mediate the conversation, which demands my full attention. It’s very convenient for the attending psychiatrist but very frustrating for the resident…. For me as the resident, it just creates more chaos” (Resident psychiatrist with more than two years of experience)

### 3.2 Evolving resident role and responsibilities

The second main theme relates to the impact of telepsychiatry on the resident’s role as a learner. This theme has two parts: first, the impact of telepsychiatry on the responsibility placed on residents, and second, the impact on their learning opportunities.

#### 3.2.1 Increased responsibility on the resident

Residents have always played a vital role in academic teaching hospitals, serving as the first point of contact for patients, conducting interviews with them and their families, and, under the supervision of the attending psychiatrist, gathering additional information to develop a comprehensive clinical picture. However, telepsychiatry has further expanded this role. In a virtual setting, residents function as key intermediaries, conveying critical details that attending psychiatrists may not have access to due to technological limitations – such as detecting strong odors or observing events that occurred before the evaluation.

“In using telepsychiatry, a resident is with the patient, so I can ask them to assess symptoms. This way, the resident can report back on what is happening” (Attending psychiatrist with 5 years or less of experience)

“The fact that it is done with the resident is what allows for a complete clinical picture” (Attending psychiatrist with 10–20 years of experience)

This new situation of remote psychiatric evaluations requires the resident to be more active and involved in the evaluation process, and to take the initiative to describe things to the attending physician that will help him or her to understand the clinical situation. Interestingly, at the interviews the residents described this change more often than the attending psychiatrists.

“The resident’s role has become much more intermediary than before” (Resident psychiatrist with more than two years of experience)

“In telepsychiatry, you don’t see the patient in full – body movements may be missed, sometimes the audio isn’t clear, or there may be internet connection issues. The attending psychiatrist doesn’t see the whole patient, for example, they can’t notice if the patient’s leg is moving off-screen. Sometimes, attendings psychiatrists assume the patient is hallucinatory, and I have to explain that telepsychiatry can create that impression. For instance, if the attending psychiatrist appeared on screen just as the patient happened to look in that direction. I have to bridge the gap, facilitate communication, and clarify what is actually being observed” (Resident psychiatrist with two years or less of experience)

“It is my responsibility to highlight things that the attending psychiatrist may not be able to see or anticipate. Ultimately, a video evaluation is only partial, as the frame captures only part of the body, not the whole person. If a patient has an injury, a specific tattoo, or an image with clinical significance – for example, a tattoo that says, ‘I will kill myself’, it is my duty to relay that information” (Resident psychiatrist with more than two years of experience)

The transition to telepsychiatry demanded effort not only from residents, but also from attending psychiatrists. The following two statements, one from an attending psychiatrist and the other from a resident, describe the extra effort needed to fully access and interpret clinical information during remote evaluations.

“Since COVID, telepsychiatry has evolved, but I feel like I’m losing something, and it takes real effort to truly connect with the patient through the screen” (Attending psychiatrist with 10–20 years of experience)

“There are patients who dissimulate and hide certain content, making it difficult to detect important details through a screen. That’s the drawback. [As the interviewer], you can’t use facial expressions or body language to subtly influence the patient and encourage them to open up – it’s much harder to do that over video call. Instead, you have to ask more questions, carefully frame the topic, and work harder to get the necessary answers. It requires extra effort” (Resident psychiatrist with more than two years of experience)

#### 3.2.2 Teaching opportunities and learning gaps

The residency period is dedicated to both teaching and learning, which takes place through various activities, including observing attending psychiatrists in practice and receiving explicit case explanations. Many participants debated whether telepsychiatry negatively impacts medical education or, conversely, introduces new learning opportunities.

Two main perspectives emerged from the interviews. Some participants believed that telepsychiatry diminishes teaching opportunities, primarily because attending psychiatrists often log off immediately after completing video evaluations, limiting further engagement and instruction. In contrast, during in-person evaluations, attending psychiatrists remain physically present in the ED, allowing for more spontaneous teaching moments and direct interaction with residents.

“If you [the attending] come to the ED, there is teaching for the resident. The residents won’t admit it, but they don’t know everything. I know because yesterday I was in the ED with a resident, and it’s clear that if I see the patient together with the resident, I will explain differential diagnoses, clinical considerations, and the benefits of hospitalization. I will guide them and open a discussion about the case – not just decide ‘yes’ or ‘no’ on involuntary hospitalization. When using telepsychiatry, the resident writes the notes on the computer, the call ends, and everyone goes their separate ways. The technology allows things to be done faster and more efficiently, but do we really want everything to be done quickly? Part of residency training is about handling acute cases, and they don’t get to see the attending psychiatrist conduct a full evaluation because I only perform a minimal assessment of the patient. In person, I would go into more depth, ask more questions, and expand the discussion” (Attending psychiatrist with 10–20 years of experience)

“I would say that telepsychiatry involves less teaching. From the few times I have used telepsychiatry – and I’m not sure if it’s directly related to telepsychiatry, [but] when the attending psychiatrist arrived in person, they were more willing to teach and explain. With telepsychiatry, it’s different. They are in a hurry to go to sleep or move on” (Resident psychiatrist with two years or less of experience)

Interestingly, some interviewees – specifically residents with at least two years of experience who had worked both before and after the introduction of telepsychiatry, argued that telepsychiatry actually enhanced teaching opportunities. They explained that during in-person evaluations, attending psychiatrists would often arrive at the ED and instruct residents to assess other patients while they conducted their own evaluations separately. However, with telepsychiatry, residents take on a crucial role as intermediaries in the attending psychiatrist’s evaluation, allowing them to stay more engaged and gain deeper insight into the attending psychiatrist’s decision-making process.

“This is not how it was before telepsychiatry, because now I have to be more involved and present during the evaluation with the attending psychiatrist. Previously, the attending psychiatrist would tell me to continue working in the ED while they conducted the evaluation. In a way, there is actually more teaching now” (Resident psychiatrist with more than two years of experience)

“For example, if there is a court order for evaluation, or a patient I believe must be hospitalized, and I have to wait for the attending psychiatrist to arrive, why should I examine them at all? It would be better for me to see another patient and wait with this one until the attending psychiatrist arrives. In that case, the patient doesn’t receive care, and I am not involved. With telepsychiatry, however, I am actively involved. I examine the patient, report my findings to the attending, and then the attending evaluates the patient via telepsychiatry” (Resident psychiatrist with more than two years of experience)

### 3.3 Changes in interactions between the attending and the resident psychiatrists

The third major theme identified in the data examines shifts in the interaction between attending and resident psychiatrists when using telepsychiatry. These changes include the heightened level of trust required for effective telepsychiatry evaluations, a more informal and relaxed communication style between attending and resident psychiatrists, and potential biases introduced by remote communication.

#### 3.3.1 Increased trust between attending and resident psychiatrists

Trust between attending and resident psychiatrists emerged as a key issue, with most participants bringing it up spontaneously, even before being directly asked. After three interviews, it was incorporated as a direct question if not already mentioned. Attending psychiatrists noted that when they have confidence in a resident’s interviewing skills, clinical judgment, and ability to conduct a thorough evaluation, they view telepsychiatry as a useful tool for making involuntary admission decisions. However, when this trust is lacking, attending psychiatrists perceive telepsychiatry as a more challenging and less reliable approach.

“There are residents I trust completely, and in those cases, telepsychiatry is an option. But compared to a real face-to-face evaluation (laughs)… the reliability isn’t high, though in some cases, it’s sufficient. If it’s a resident I trust and the case is clear, it works. Anything else, I don’t rely on it” (Attending psychiatrist with more than 20 years of experience)

“There are some highly experienced resident physicians, and I trust their judgment … However, in certain cases where I don’t fully trust a resident or their clinical judgment, I would prefer to come to the ED myself” (Attending psychiatrist with 10–20 years of experience)

“In medicine, this happens all the time – you examine a patient, they say X, and then the attending asks, and suddenly it’s Y. Like any attending psychiatrist, even before telepsychiatry, they heavily relied on the resident’s report – very, very much. Some attending psychiatrists trust the resident completely and don’t obsess over details, while others ask a million questions and don’t rely on a more superficial evaluation” (Resident psychiatrist with more than two years of experience)

Most residents reported feeling trusted by the attending psychiatrist, with no noticeable change in trust levels before or after the introduction of telepsychiatry. As one resident observed, the level of trust a resident receives is largely dependent on their seniority and experience.

“I think it depends on the resident. If the resident is more experienced, their evaluation will be more thorough, making the attending psychiatrist’s evaluation more effective. The attending psychiatrist will still conduct his own evaluation, but he will listen more and be less likely to miss important details” (Resident psychiatrist with more than two years of experience)

It is important to note that attending and resident psychiatrists are already familiar with each other from working together in hospital departments or the ED. This existing face-to-face experience may influence the level of trust between them.

#### 3.3.2 More relaxed interpersonal communication

Residents, especially those who had worked in the ED before telepsychiatry was introduced, observed that telepsychiatry fosters a more relaxed and less tense interaction between them and the attending psychiatrists. Attending psychiatrists recognize that successful hospitalization and treatment outcomes improve when patients provide consent, so they guide residents in persuading patients to agree to admission.

Previously, when patients refused hospitalization, attending psychiatrists had to be physically present – sometimes multiple times per shift and after long drives, to either convince the patient or, as a last resort, authorize involuntary admission. With telepsychiatry, attending psychiatrists can still apply their persuasive skills remotely and, if necessary, make involuntary hospitalization decisions via video call. This reduces logistical strain while allowing them to remain actively involved in decision-making.

Additionally, remote consultation alleviates a potential source of pressure for residents, as they can pursue what they believe is in the patient’s best interest without worrying about summoning the attending psychiatrist to the hospital in the middle of the night.

“It eases the burden on both the attending psychiatrists and the residents because it’s really unpleasant to wake up the attending psychiatrist and ask them to come to the ED at 2, 3, and 4 AM. It’s an uncomfortable situation at night, but with telepsychiatry, it’s different” (Resident psychiatrist with more than two years of experience)

#### 3.3.3 Concerns about increased opportunities for bias with remote evaluation

As previously discussed, telepsychiatry places residents in a more intermediary role, requiring them to relay crucial information that attending psychiatrists may not have direct access to due to technological limitations. Some attending psychiatrists expressed concern that relying on residents to convey key details could introduce bias into clinical decision-making.

“I think that when the resident seeks consultation to make a decision and values a quick response, they may push for and encourage telepsychiatry. However, we need to be honest with ourselves, as this method does not always allow for the highest level of accuracy. Sometimes, the case is not clear-cut, and telepsychiatry doesn’t necessarily help – it only highlights the gaps. You can’t see everything through a screen, and there’s a risk of missing something crucial for decision-making. If you compromise too much, you may end up compromising your own professional standards as well” (Attending psychiatrist with 10–20 years of experience)

“The attending psychiatrist relies more on the resident’s impression. I found that when I had fewer sensory cues, I depended more on hearing the resident’s evaluation, which helped orient me and guide my approach before even evaluating the patient. However, it was not the same as conducting the evaluation myself. The evaluation felt less reliable, though in some cases, it was still sufficient for issuing an involuntary hospitalization order” (Attending psychiatrist with more than 20 years of experience)

However, other attending and resident psychiatrists disagreed, arguing that telepsychiatry does not introduce bias or prevent attending psychiatrists from forming an objective impression of the patient. Instead, they maintained that telepsychiatry evaluations remain independent of resident input and that there is no inherent bias in the process.

## 4 Discussion

### 4.1 Discussion

This study aimed to explore attending and resident psychiatrists perceive the impact of telepsychiatry on communication and interactions both between the psychiatrists and patients and between the attending and resident psychiatrists in the ED, specifically in the context of considering possible involuntary evaluations. Through qualitative approaches using interviews with attending and resident psychiatrists in a large ED, we identified three key themes: shifts in communication patterns between physicians and patients, the evolving role of residents with increased responsibility and teaching opportunities, and changes in the attending–resident psychiatrist dynamic in the context for remote evaluation.

Our findings suggest that telepsychiatry, when used while the resident and the patient are in the ED and the attending psychiatrist is elsewhere, can have a meaningful impact on psychiatrist–patient communication. A central concern expressed by many participants was whether telepsychiatry evaluation compromises professionalism and the ability to establish a strong therapeutic connection with patients. While telepsychiatry was viewed by most participants as an efficient and practical tool in the ED, others questioned whether it fully aligns with the traditional standards of medical professionalism. Similar concerns have been documented in other studies. For example, in 2021 US survey of 819 healthcare providers found that while the majority viewed telepsychiatry positively due to improved scheduling flexibility and reduced delays, nearly half felt less connected to patients compared to in-person interactions (53). These concerns highlight the need for evidence-based strategies to uphold professional standards and ensure that telepsychiatry is adapted effectively across different settings, including emergency services and involuntary admission contexts.

Our study also revealed that telepsychiatry, when used for potential involuntary hospitalizations where the resident and the patient are in the hospital and the attending psychiatrist is elsewhere, has expanded the responsibilities of residents, particularly in cases involving involuntary hospitalization. Using telepsychiatry, residents serve as intermediaries, relaying crucial clinical information that attending psychiatrists cannot directly observe via video, such as sensory cues or patient behavior prior to the evaluation. Given the limited research on the role of telepsychiatry in ED settings, especially for involuntary hospitalization, these findings represent an important contribution of the field.

In context of how telepsychiatry was used in this study, our participants expressed mixed opinions regarding how it had impacted residents’ education. While some viewed it as a barrier to traditional teaching, others believed it created new learning opportunities by increasing resident involvement in the evaluation and decision-making process. The rapid expansion of telehealth in emergency medicine, including for triage and virtual observation, has outpaced curriculum development in residency training programs (44). Although early evidence indicates that telemedicine training can be effectively integrated into residency education (40, 41), it is much less common for training programs to address its use in emergency settings. We only found one study of the use of telehealth in an ED setting (44), and none addressing the use of telepsychiatry in the ED for involuntary cases. This gap highlights the need for further research to develop structured, specialty-specific programs that enhance telepsychiatry for the evaluation of patients for potential involuntary admission. Therefore, there is a need for both research and educational efforts to examine the effectiveness of telepsychiatry for this indication, and to teach doctors how to use it most effectively.

In the setting where telepsychiatry is employed for involuntary hospitalization evaluations, with the resident and patient present in the hospital while the attending psychiatrist engages remotely, another key concern raised by participants was the potential for bias in remote clinical decision-making. This aligns with existing research on cognitive bias in telemedicine. For example, Haimi et al. (54) examined 15 pediatric telehealth consultants and found that physicians’ clinical decisions were influenced by non-medical factors such as parental characteristics or communication styles. In a separate study, the same research group conducted a retrospective analysis of 339 randomly selected parent–physician telemedicine consultations via phone calls between 2014 and 2017. The study examined how non-medical factors influenced primary and secondary clinical decisions, diagnostic accuracy, and the “reasonability” of decisions. Findings suggested that various non-medical factors, including child-related characteristics and the background of the parent speaking to the physician, significantly shaped physicians’ decisions, demonstrating the influence of bias in telemedicine settings (55). These studies focused on phone consultations rather than live-video telepsychiatry. Both phone and video telepsychiatry have proven effective compared to in-person evaluations, with preferences based on specific psychiatric needs (56, 57). For example, a review by Chen et al. (57) found that both phone and video treatments reduce mental health symptoms and are non-inferior to in-person care. While video provides valuable visual information for diagnosis, phone consultations are more accessible and tend to have fewer technological issues. Given the access barriers for certain populations who may only have audio-only options, it is important to recognize that unintended bias can occur in any evaluation setting, even in-person, but may be heightened in remote telemedicine, with video communication and even more so with audio-only communication.

Additionally, research in telemedicine education suggests that clinicians who receive more background information on a patient may be prone to anchoring bias, in which early details disproportionately influence their diagnostic reasoning. A recent study has introduced another dimension of bias in telemedicine decision-making. This study examined 72 medical students to assess how exposure to varying amounts of background and medical information about patients affected their clinical decision-making. Using an online questionnaire with medical case vignettes, the study simulated asynchronous telemedicine settings where clinicians assess cases electronically without real-time patient interaction. Results showed that students who received more information tended to rely excessively on initial details. However, training interventions successfully mitigated this anchoring bias, particularly among students with strong cognitive abilities and quick decision-making skills (58). Currently, there is a lack of research specifically addressing clinical reasoning and bias in telepsychiatry decision-making. Further investigation is needed to determine whether remote psychiatric evaluations impact clinical reasoning and, if so, how best to address this.

### 4.2 Limitations

This study has several limitations. As this study was conducted at a single site, where both the resident and the patient were in the hospital and the attending psychiatry joined remotely from another location to consider possible involuntary admission, the generalizability of the findings may be limited. For example, in settings where there is no psychiatry resident, and an emergency physician has seen the patient before the psychiatrist begins the video-call, the results could be different. However, given the scarcity of research on telepsychiatry in ED settings, these findings provide valuable initial insights. Second, while the CanMEDS framework was applied in the analysis, it was not explicitly incorporated into the interview guide, meaning that participants were not directly asked about these concepts. As a result, it is unclear whether additional insights would have emerged had the framework been introduced earlier. Additionally, due to insufficient data, we were unable to systematically compare the perspectives of attending and resident psychiatrists regarding changes in doctor-patient communication, which limits the ability to draw meaningful distinctions between these two groups. Nevertheless, CanMEDS proved useful in structuring the findings and identifying areas for further exploration. Finally, this study focused solely on the perspectives of psychiatrists, and patient perspectives were not included. While clinician-driven decisions are central to involuntary admissions, future research should incorporate patient experiences to provide a more comprehensive understanding of how telepsychiatry affects psychiatric care.

### 4.3 Future directions

Further research is needed to examine the impact of telepsychiatry across different healthcare settings, cultural contexts, and regulatory environments. Cross-cultural studies could provide insights into how telepsychiatry influences physician–patient and attending–resident psychiatrist dynamics in diverse healthcare systems. Additionally, future research should include structured patient-reported outcomes to assess satisfaction, trust, and engagement in remote psychiatric evaluations compared to in-person care. Understanding patient perspectives will be critical in refining telepsychiatry practices and ensuring that remote care remains patient centered. It is essential to establish a clear consensus on supervisee–supervisor roles and expectations when delivering care via telepsychiatry, as this supports effective collaboration and safeguards the quality of both training and patient care. In addition, there is a pressing need to develop structured training programs for telepsychiatry, with particular attention to the unique demands of emergency psychiatry and involuntary hospitalizations. Training efforts should focus on optimizing communication strategies, minimizing bias in remote assessments, and ensuring that residents receive adequate supervision in psychiatric settings. By improving resident–attending psychiatrist interactions and enhancing clinical decision-making, such initiatives can help maintain professionalism and ensure high-quality, equitable care in telepsychiatry.

### 4.4 Conclusions

This study examined how the transition to telepsychiatry, replacing in-person evaluations by attending psychiatrists, affected communication dynamics in the psychiatric ED, and specifically for possible involuntary admissions. Using qualitative interviews with attending and resident psychiatrists, we identified three key themes: changes in psychiatrist–patient communication, the evolving role of residents – including increased responsibility and expanded learning opportunities and shifts in the attending–resident dynamic. Our findings underscore the lack of formal training and structured guidelines for telepsychiatry, emphasizing the urgent need for organized education prior to its use by both supervisors and supervisors, together with clear agreement on their respective roles. As telepsychiatry continues to expand, ongoing evaluation and evidence-based policy development will be critical in optimizing its implementation, uphold high standards of patient-centered psychiatric care, and minimize potential biases.

## Data Availability

The original contributions presented in the study are included in the article/**Supplementary Material**. Further inquiries can be directed to the corresponding author.
