# Supplementary material for: Click to consult: psychiatrists’ perspectives on how telepsychiatry impacts communication in the emergency department
Source: Front Psychiatry. 2025 Sep 5;16:1629475. doi: 10.3389/fpsyt.2025.1629475 (PMC12447525; doi:10.3389/fpsyt.2025.1629475)
Supplement: Supplementary file 1 [file Table1.docx]

**Semi-structured interview guide- Click to consult: Psychiatrists' perspectives on how telepsychiatry impacts communication in the emergency department (ED)**

| Topic | Questions |
| --- | --- |
| Background Information | 1. Can you please tell me about yourself (age, current role in the ED, how long you have been working in the ED specifically and in psychiatry in general, area of expertise in psychiatry). |
| Perceptions about telepsychiatry | 2. Please describe your experience with telepsychiatry in the ED so far.  3. What is the typical flow of using telepsychiatry in the ED?  4. What are your general perceptions about the use of telepsychiatry in the ED setting for possible involuntary hospitalizations?  5. When compared to in-person evaluations, what are the advantages and disadvantages of using telepsychiatry (In relation to  the patient, the flow of the ED, the resident and the attending psychiatrist)?  6. What challenges do you see with using telepsychiatry for psychiatric evaluations in the ED for possible involuntary hospitalizations?  7. In your opinion, in what way does telepsychiatry provide quality of evaluation compared to in-person evaluations in involuntary cases? |
| Impact on communication and interaction between residents and attending psychiatrists | For residents who experienced only telepsychiatry modality during the evening and night shifts:  8. Please describe the way you communicate with the attending psychiatrist when telepsychiatry is involved  For attending psychiatrists and attendings which experienced in-person and telepsychiatry modalities during the evening and night shifts:  8. How has the introduction of telepsychiatry changed the way you communicate with the attending/resident psychiatrist?  9. Have you noticed any specific communication challenges or improvements?  10. How does telepsychiatry affect the exchange of information during patient evaluations?  11. How involved do you feel during telepsychiatry evaluations? Has your level of involvement changed when using telepsychiatry compared to in-person evaluations?  12. Have there been any changes in your roles or responsibilities as a resident when using telepsychiatry versus in-person evaluations? |
| General thoughts and suggestions for improvement | 13. What would you change or improve regarding the use of telepsychiatry in the ED for involuntary cases? Are there any technical or operational improvements you would suggest? Are there any specific areas where telepsychiatry could be more beneficial? |
| Closing | 14. Is there anything else you would like to share about your experience with telepsychiatry in the ED? |
